# Side-by-side comparison of recombinant human glutathione peroxidases identifies overlapping substrate specificities for soluble hydroperoxides

**DOI:** 10.1016/j.redox.2022.102593

**Published:** 2023-01-02

**Authors:** Maria Schwarz, Alina Löser, Qing Cheng, Mareike Wichmann-Costaganna, Patrick Schädel, Oliver Werz, Elias SJ. Arnér, Anna P. Kipp

**Affiliations:** a Department of Nutritional Physiology, Institute of Nutritional Sciences, Friedrich Schiller University Jena, Jena, 07743, Germany; b TraceAge-DFG Research Unit on Interactions of Essential Trace Elements in Healthy and Diseased Elderly, Potsdam-Berlin-Jena, Germany; c Division of Biochemistry, Department of Medical Biochemistry and Biophysics, Karolinska Institutet, SE-171 77, Stockholm, Sweden; d Department of Pharmaceutical/Medicinal Chemistry, Institute of Pharmacy, Friedrich Schiller University Jena, Germany; e Department of Selenoprotein Research, National Institute of Oncology, 1122, Budapest, Hungary

**Keywords:** Glutathione peroxidase, Selenoprotein, Hydroperoxides, CHP, cumene hydroperoxide, GPX, glutathione peroxidase, HpETE, hydroxy-eicosatetraenoic acid, HpODE, hydroxy-octadecadienoic acid, MEF, mouse embryonic fibroblast, PCOOH, phosphatidylcholine hydroperoxide, Sec, selenocysteine, tBHP, *tert*-butyl hydroperoxide

## Abstract

Five out of eight human glutathione peroxidases (GPXs) are selenoproteins, representing proteins that contain selenium as part of the amino acid selenocysteine. The GPXs are important for reducing hydroperoxides in a glutathione-consuming manner and thus regulate cellular redox homeostasis. GPX1, GPX2, and GPX4 represent the three main cytosolic GPXs, but they differ in their expression patterns with GPX1 and GPX4 being expressed ubiquitously, whereas GPX2 is mainly expressed in epithelial cells. GPX1 and GPX2 have been described to reduce soluble hydroperoxides, while GPX4 reduces complex lipid hydroperoxides, thus protecting cells from lipid peroxidation and ferroptosis. But most of these data are derived from cells that are devoid of one of the isoforms and thus, compensation or other cellular effects might affect the conclusions. So far, the use of isolated recombinant human selenoprotein glutathione peroxidases in pure enzyme assays has not been employed to study their substrate specificities side by side. Using recombinant GPX1, GPX2, and GPX4 produced in E. coli we here assessed their GPX activities by a NADPH-consuming glutathione reductase-coupled assay with 17 different peroxides (all at 50 μM) as substrates. GPX4 was clearly the only isoform able to reduce phosphatidylcholine hydroperoxide. In contrast, small soluble hydroperoxides such as H_2_O_2_, cumene hydroperoxide, and *tert*-butyl hydroperoxide were reduced by all three isoforms, but with approximately 10-fold higher efficiency for GPX1 in comparison to GPX2 and GPX4. Also, several fatty acid-derived hydroperoxides were reduced by all three isoforms and again GPX1 had the highest activity. Interestingly, the stereoisomerism of the fatty acid-derived hydroperoxides clearly affected the activity of the GPX enzymes. Overall, distinct substrate specificity is obvious for GPX4, but not so when comparing GPX1 and GPX2. Clearly GPX1 was the most potent isoform of the three GPXs in terms of turnover in reduction of soluble and fatty-acid derived hydroperoxides.

## Highlights

• Isolated recombinant human glutathione peroxidases are compared side by side.
• GPX4 is the only isoform able to reduce phosphatidylcholine hydroperoxide.
• Small soluble and fatty acid-derived hydroperoxides are reduced by GPX1, 2 and 4.
• No substrate specificity has been identified for GPX1 and GPX2.
• GPX1 was the most potent isoform of the three GPXs in terms of substrate turnover.

## 1 Introduction

In mammalian cells, reactive oxygen species, such as H_2_O_2_ or fatty acid-derived hydroperoxides, are generated continuously during aerobic metabolism and in some enzymatic reactions e.g. catalysed by NADPH oxidases, ERO1, glucose or xanthine oxidases, and cyclooxygenases or lipoxygenases during eicosanoid biosynthesis. While these short-lived compounds can act as important signalling molecules modulating redox-sensitive signalling cascades, too high concentration can have detrimental effects for the cell. To prevent damage caused by peroxide overload, they have to be eliminated which is mainly mediated by antioxidant enzymes [1]. There are several enzymes capable of peroxide reduction including catalase, peroxiredoxins, and glutathione peroxidases (GPXs) [2]. Among those, the GPX family is special because five (GPX1–4 and 6) of its eight members are selenoproteins in humans, meaning that they contain a selenocysteine (Sec) residue [3]. A common feature of the GPX enzymes is a conserved tetrade with the peroxidatic Sec as one of the catalytically active amino acids in addition to glutamine, tryptophan, and asparagine [4,5]. During the catalytic cycle, the Sec residue is oxidized by hydroperoxides forming selenenic acid or a selenenylamide intermediate, which are then reduced back to selenolate by thiols, in most cases by glutathione [6,7]. As Sec is a highly reactive amino acid, it is supposed to be important for maximizing the activity of GPX enzymes towards hydroperoxides. There are several hypothesis regarding the advantage of having Sec instead of cysteine which have been discussed before [8,9]. Accordingly, especially GPXs are believed to be of major importance for maintaining redox balance.

GPX1 was the first selenoprotein identified [10,11] and with this finding the area of selenoprotein research emerged. For the GPX enzymes it eventually became clear that they differ in their localization and potentially in their substrate specificities. While GPX1 and GPX4 are ubiquitously expressed, GPX2 is specifically located in epithelial cells, and GPX6 is only expressed in the olfactory epithelium. In contrast, GPX3 is an extracellular protein which contributes to the selenium concentration in plasma but is also detectable in other extracellular body fluids such as chamber water of the eye, thyroid colloid lumen or amniotic fluid [12]. GPX1 is localized in the cytosol as well as in mitochondria [13], reacts most sensitive towards changes of the selenium supply and is substantially downregulated during deficiency [14,15]. Its hepatic expression is discussed to serve as the body storage of selenium. GPX4, also called phospholipid hydroperoxide GPX, differs from the other GPXs in that it can reduce phospholipid and cholesteryl hydroperoxides bound to cell membranes, and thus protects biomembranes from oxidation [16,17]. The lipid bound hydroperoxides only become accessible to GPX1 when membranes are pre-incubated with phospholipase A_2_ to liberate fatty acid hydroperoxides [18]. There are three different GPX4 isoforms which are located in the cytosol, mitochondria, nucleus, or at the plasma membrane. The cytosolic GPX4 is not only located in the cytosol but also in membrane compartments in a broad spectrum of cell types but predominantly in neurons. Mitochondrial and nuclear GPX4 are almost exclusively expressed in testes [19]. So far, GPX6 has only been described to be localized in the olfactory epithelium [20].

The physiological role of the intracellular selenoproteins GPX1, 2, and 4 for regulating the cellular redox status has been studied and described in genetically modified cells and mice [21,22]. Embryonic lethality has been observed for a systemic knockout of the entire GPX4 gene [23] or for a knockout of cytosolic GPX4 [24]. Conditional GPX4 knockout mice revealed that GPX4 is essential for maintaining tissue homeostasis of several organs including brain, skin, and endothelium [25,26]. Cells without GPX4 are very sensitive towards ferroptosis mediated by an overwhelming lipid peroxidation [27]. In contrast to this severe phenotype, effects of genetic ablation of either GPX1 or GPX2 are less pronounced. Under basal conditions, no obvious phenotypes have been detected for either GPX1 or GPX2 knockout mice [[28], [29], [30]]. When GPX1 knockout mice are treated with redox cyclers or bacterial endotoxins to trigger H_2_O_2_ production, however, they are severely affected indicating that GPX1 is important to protect many cell types and tissues from oxidative stress [31,32]. GPX2 knockout mice show enhanced cell death in the intestinal stem cell compartment with concomitant modification of the differentiated epithelial cell populations [33,34]. In addition, more intraepithelial inflammatory cells can be found, indicating that loss of GPX2 modulates the intestinal stem cell niche [35]. Next to intestinal stem cells, also many types of tumour cells show upregulation of GPX2 supporting the view that GPX2 expression is specifically enhanced in highly proliferating cells [36].

Except for their differences in localization, it is still not entirely clear why the two different isoforms GPX1 and 2 coexist, and whether they differ in their selectivity and/or efficiency to reduce specific hydroperoxide species. Initial experiments characterizing GPX1 and 2 used MCF-7 cells which do not express endogenous GPX1 or 2 and, accordingly, stably overexpressing cell lines were generated for either GPX1 or GPX2 [37]. Enzyme preparations from the two overexpressing lines had substantially higher total GPX activity towards H_2_O_2_, *tert*-butyl hydroperoxide (tBHP), cumene hydroperoxide (CHP), and linoleic acid hydroperoxide, in comparison to the ones from wild type MCF-7 cells. It was thus suggested that GPX1 and GPX2 have similar substrate specificities [37]. However, these initial assumptions were limited by the cellular system used because proper selenoprotein overexpression is difficult to achieve in mammalian cell systems; even if their expression levels are improved by genetic methods the yields are still too low for practical use in isolation of the pure selenoproteins [38]. Experiments with isolated proteins have thus mainly been using GPX variants purified from mammalian tissues such as porcine or bovine liver or human erythrocytes [6]. Until recently, the generation of recombinant human selenoproteins in bacterial systems was very difficult, especially for selenoproteins with the Sec residue located further from the C-terminus. However, a novel system now enables expression and purification of such recombinant selenoproteins, including human GPX enzymes [5,39,40]. Herein, we used such recombinantly expressed and purified human selenoproteins GPX1, GPX2, and GPX4, enabling for the first time side by side comparisons in order to reveal their individual activities with a set of 17 different hydroperoxides.

## 2 Methods

### 2.1 Preparation and purification of recombinant proteins

The GPX1, 2 and 4 recombinant proteins were prepared as previously described [39] and where kept at -20°C in 50 mM Tris-HCl (pH 7.5) with 100 mM NaCl, 5 mM β-mercaptoethanol and 20% glycerol as concentrated stock aliquots until use. On SDS-PAGE the protein preparations were apparently homogenously >98% pure, but as they have less than 100% Sec contents due to non-Sec suppression events (see below) the selenium contents were determined for normalisation of Sec-dependent turnover values, as described next and in the Results section.

### 2.2 Determination of selenium

The selenium content of the recombinant proteins stored in buffer (50 mM Tris-HCl, pH 7.5, 100 mM NaCl, 5 mM β-mercaptoethanol, and 20% glycerol) was determined using a bench-top total reflection X-ray fluorescence (TXRF) spectrophotometer (TSTAR, Bruker Nano, Berlin, Germany) with 1 mg/l gallium (Merck/Millipore, Burlington, MA, USA) as internal standard. 16 μl of each sample was mixed with 1.6 μl gallium standard and 3 μl of this solution were prepared on five siliconized sample carriers and measured for 1,000 s. To calculate % selenium content of the recombinant proteins, the obtained selenium concentration was normalized to total protein concentration which was determined by absorbance of the pure proteins at 280 nm and using the calculated extinction coefficient for each protein at 280 nm as based upon the respective amino acid sequence.

### 2.3 Generation and cultivation of mouse embryonic fibroblast (MEF) cells

The GPX1/GPX2 double knockout mice were kindly provided by Steven Esworthy and Fong-Fong Chu [41]. Embryos were isolated from pregnant mice at E13.5. The body trunk was dissected away from other structures and then treated with trypsin and with DNase (Roche, Mannheim, Germany). The cell suspension was filtered and resuspended in fresh medium. MEF cells were cultured under standard culture conditions (37°C, 5% CO_2_) in Roswell Park Memorial Institute 1640 medium (RPMI; ThermoFisher Scientific, Waltham, USA) supplemented with 10% fetal calf serum (Sigma-Aldrich/Merck, Taufkirchen, Germany), 1% penicillin-streptomycin (ThermoFisher Scientific), 1% GlutaMAX^TM^ (ThermoFisher Scientific), and 50 nM sodium selenite in cell culture dishes coated with poly-L-lysin (Sigma-Aldrich/Merck). Cells were harvested to generate protein lysates for GPX activity measurements or for RNA isolation. For genotyping of mice, tail biopsies were lysed in alkaline buffer (25 mM NaOH, 0.2 mM EDTA, pH 12) at 95°C for 1 h. After neutralisation with the equal amount of neutralisation buffer (40 mM Tris, 0,04% HCl, pH 3), 1 μl of the lysate was used as template for the subsequent PCR. The PCR was carried out over 40 cycles with DreamTaq Green Polymerase (ThermoFischer Scientific) and oligonucleotides corresponding to [41].

### 2.4 Western Blot

Recombinant protein (500 ng of each preparation) was loaded on a 15% acrylamide gel followed by SDS polyacrylamide gel electrophoresis. After immunoblotting of the proteins to a nitrocellulose membrane, protein transfer was controlled after a 2 min shaking step of the membrane in Ponceau S solution (0.2% Ponceau S (Carl Roth, Karlsruhe, Germany) with 3% (w/v) trichloracetic acid (Carl Roth)) and bands were recorded with the ChemiDoc^TM^ MP Imaging system (Bio-Rad, California, USA). Afterwards membranes were incubated in 5% non-fat dry milk (NFDM) powder diluted in Tris buffered saline containing 0.1% Tween 20 (TTBS) for 1 h at room temperature. Membranes were cut into three parts which were incubated with either rabbit anti-GPX1 (epitomics; 3120-1; 1:2000), rabbit anti-GPX2 (GBF, [42]) or rabbit anti-GPX4 (abcam; 125066; 1:2000) diluted in TTBS overnight at 4 °C, respectively. As secondary antibody the goat anti-rabbit horseradish peroxidase-conjugated antibody (cell signaling #7074S; 1:50,000) diluted in 5% NFDM-TBST was used. By using the SuperSignal^TM^ West Dura (ThermoFisher Scientific) band intensities could be imaged in the ChemiDoc^TM^ MP Imaging system.

### 2.5 GPX activity measurement

The GPX activity was measured as previously described [33,43,44] in a NADPH-consuming glutathione reductase (GR) coupled assay. MEF cells were lysed in Tris buffer (100 mM Tris, 300 mM KCl (Applichem, Darmstadt, Germany), 0.1% (v/v) Triton X-100 (Carl Roth)) using the TissueLyser II (Qiagen, Hilden, Germany) with two homogenizing steps at 30 Hz each for 1 min. Afterwards, cellular debris was removed by centrifugation (14,000×*g*, 10 min, 4°C). Samples were mixed with reaction mixture (85 mM Tris, 1.4 nM EDTA, 0.85 NaN_3_ (Sigma-Aldrich/Merck), 0.1% Triton X-100, 0.2 mM NADPH (Carl Roth), 3 mM GSH (Sigma-Aldrich/Merck) and 0.07 U/ml GR (Sigma-Aldrich/Merck) and incubated for 15 min at 30°C. The GPX reaction was initiated by adding 10 μl of each 750 μM substrate solution. The NADPH consumption was measured over 5 min at 30°C and 340 nm using a microplate reader (Synergy H1, Biotek). For the MEF cell lysates, hydrogen peroxide (H_2_O_2_, Sigma-Aldrich/Merck), *tert*-butyl hydroperoxide (tBHP, Sigma-Aldrich/Merck), and the fatty acid derived hydroperoxides hydroxy-octadecadienoic acid (HpODE), and hydroxy-eicosatetraenoic acid (HpETE) [45] were used as substrates. The GPX activities of the MEF cell samples were normalized to protein contents of the cell lysates which were analysed by Bradford assay.

The recombinant GPX enzymes were analysed with a broader substrate spectrum including H_2_O_2_, tBHP, cumene hydroperoxide (Sigma-Aldrich/Merck), phosphatidylcholine hydroperoxide (PCOOH, [44]) and specific fatty acid-derived hydroperoxides listed in Table 1. All of the fatty acid-derived hydroperoxides were evaporated and a 3.75 mM stock was prepared using 98% ethanol (Carl Roth). For the measurement, the hydroperoxides were diluted to a final concentration of 750 μM with aq. dest.. For all substrates a final concentration of 50 μM was used in the assay. The amount of ethanol in the assay did not affect the outcome. To measure the GPX activity with the recombinant selenoproteins, 10 nM GPX1, 115 nM GPX2 and 120 nM GPX4 (48 nM GPX4 for PCOOH) were used because these protein concentrations resulted in a linear reaction for at least 2 min. Prior to calculation of enzymatic activity, the activity curves (decrease in absorbance at 340 nm over time) were corrected so that only the linear parts of the curves were included in the analyses. The corresponding ΔA^340^/min was used for calculations after the subtraction of the minor ΔA^340^/min received from a blank containing all assay components except for the GPX enzyme. Further calculations were: ΔE/min × 18.75 (dilution factor: 8 μl enzyme in 150 μl total volume)/6.22 (mmol/L)^−1^ × cm^−1^ (ϵ = extinction coefficient for NADPH). Consumption of 1 μmol NADPH/min was taken as 1 U. The absorbance reader corrects filling levels to a light path length of 1 cm. This way, activity can be calculated according to Lambert–Beer's law. The respective activity was calculated in relation to the applied protein concentration for each GPX isoform (10 nM GPX1, 115 nM GPX2 or 120 nM GPX4) considering only the amount of active selenoprotein (20% for GPX1, 14% for GPX2 and 13% for GPX4). This results in the specific activities which were finally converted to U per mg selenoprotein GPX enzyme.

**Table 1:** Fatty acid derived hydroperoxides purchased from Cayman chemicals.

Fatty acid	Hydroperoxides		Order No.
	full name	abbreviation	
Octadecadienoic acid (Linoleic acid)	(±)9-hydroperoxy-10E,12Z-octadecadienoic acid	(±)9-HpODE	Cay1070
	9S-hydroperoxy-10E,12Z-octadecadienoic acid	9(S)-HpODE	Cay48410
	(±)13-hydroperoxy-9Z,11E-octadecadienoic acid	(±)13-HpODE	Cay10704
	13S-hydroperoxy-9Z,11E-octadecadienoic acid	13(S)-HpODE	Cay48610
Eicosatetraenoic acid (Arachidonic acid)	5S-hydroperoxy-6E,8Z,11Z,14Z-eicosatetraenoic acid	5(S)-HpETE	Cay44230
	12-hydroperoxy-5Z,8Z,10E,14Z-eicosatetraenoic acid	(±)12-HpETE	Cay10138
	12S-hydroperoxy-5Z,8Z,10E,14Z-eicosatetraenoic acid	12(S)-HpETE	Cay44570
	15S-hydroperoxy-5Z,8Z,11Z,13E-eicosatetraenoic acid	15(S)-HpETE	Cay44720
Eicosapentaenoic acid	15S-hydroperoxy-5Z,8Z,11Z,13E,17Z-eicosapentaenoic acid	5(S)-HpEPE	Cay42210
	12(S)-hydroperoxy-5Z,8Z,10E,14Z,17Z-eicosapentaenoic acid	12(S)-HpEPE	Cay42550
	15S-hydroperoxy-5Z,8Z,11Z,13E,17Z-eicosapentaenoic acid	15(S)-HpEPE	Cay42710
Eicosadienoic acid	15S-hydroperoxy-11Z,13E-eicosadienoic acid	15(S)-HpEDE	Cay47720
Docosahexonoic acid	17S-hydroperoxy-4E,7Z,10Z,13Z,15Z,19Z-docosahexanoic acid	17(S)-HpDHA	Cay13185

### 2.6 Mass spectrometry-based analysis of monohydroxylated fatty acids after GPX reaction

GPX conversion of fatty acid derived hydroperoxides was performed as mentioned before. The reaction was stopped by addition of 1 mL ice-cold MeOH (fisher chemical; 10653963) and samples were directly processed for measurement. Therefore, solid phase extraction was performed after initial acidification (Milli-Q water, pH 3.5) using SPE cartridges (Waters, WAT043395). Samples were washed with 6 mL of Milli-Q water and eluted with 6 mL of methyl formate (fisher chemical; 414340025) into a glass vial. Samples were evaporated until dryness with a moderate stream of nitrogen and resuspended in 100 μL of an equal mixture of MeOH and water (VWR Chemicals, 83645320). Monohydroxylated fatty acids were measured by ultra-high-performance liquid chromatography (UPLC) tandem mass spectrometry in line with previously published methodology [46].

### 2.7 Quantitative real-time-PCR (qPCR)

The mRNA was isolated using the Dynabeads mRNA DIRECT Kit (Fisher Scientific) according to the manufacturer's protocol. Using 150 fmol oligo (dT) 15 primers and 180 U Moloney Murine Leukemia Virus Reverse Transcriptase (M-MLV RT; Promega, Mannheim, Germany), 100 ng mRNA were transcribed into cDNA by reverse transcriptase PCR. Real-time PCR was performed in a total volume of 25 μL with 1 μL of diluted cDNA and SYBR Green 1 (Molecular Probes, Eugene, USA) as fluorescent reporter using a Mx3005P™ qPCR system (Agilent). Standard curves from diluted PCR products were used for quantification. cDNA-specific primers were used: murine Gpx1: (NM_008160); fwd GAAGAGATTCTGAATTCCCTCAA; rev CACACCAGGAGAATGGCAAGA; murine Rpl13a (NM_009438.5); fwd: GTTCGGCTGAAGC CTACCAG; rev: TTCCGTAACCTCAAGATCTGCT (Sigma-Aldrich). Rpl13a was used as reference gene.

### 2.8 Statistics

Values are presented as mean +SD. The Student's t-test (unpaired, two-tailed) was performed to analyse two groups, one-way ANOVA for comparing more than two groups, and two-way ANOVA for two-parametric data with Bonferroni's post-hoc test using Graphpad Prism 8. Differences with a p value of less than 0.05 were considered statistically significant.

## 3 Results

### 3.1 Murine embryonic fibroblasts with knockout of either GPX1 or GPX2 display substantially altered total cellular GPX activities

**Fig. 1:**
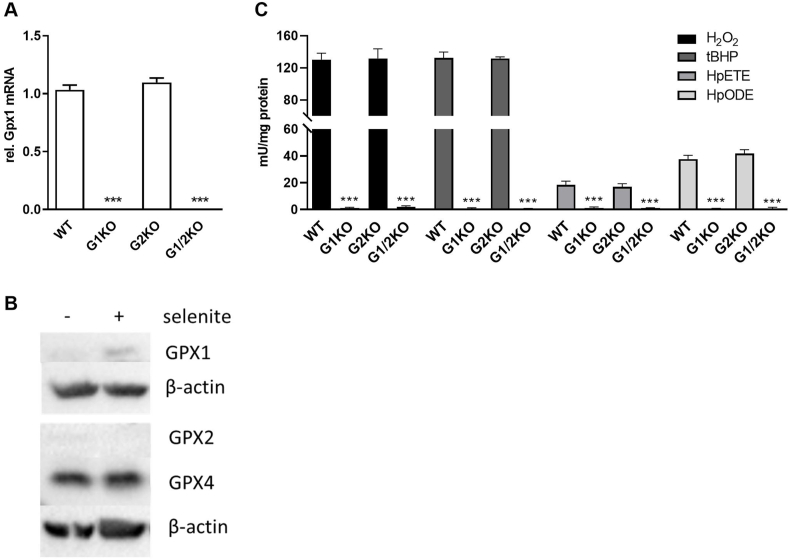
**In murine embryonic fibroblasts, total GPX activity is mainly catalysed by GPX1.** Murine embryonic fibroblasts were cultivated with 50 nM sodium selenite. The mRNA expression of GPX1 was analysed by qPCR and normalized to Rpl13a (A). Representative Western Blots showing GPX1, GPX2, and GPX4 expression depending on the selenium supply using β-actin as reference protein (B). GPX activity was measured photometrically by a GR-coupled test using 50 μM H_2_O_2_, tBHP, HpETE, and HpODE as substrates and normalized to protein concentration (C). Data are depicted as mean + SD (n = 3). Statistical analyses were based on one-way ANOVA with Bonferroni’s post-test. ***p < 0.001 vs. WT.

**Figure S1:**
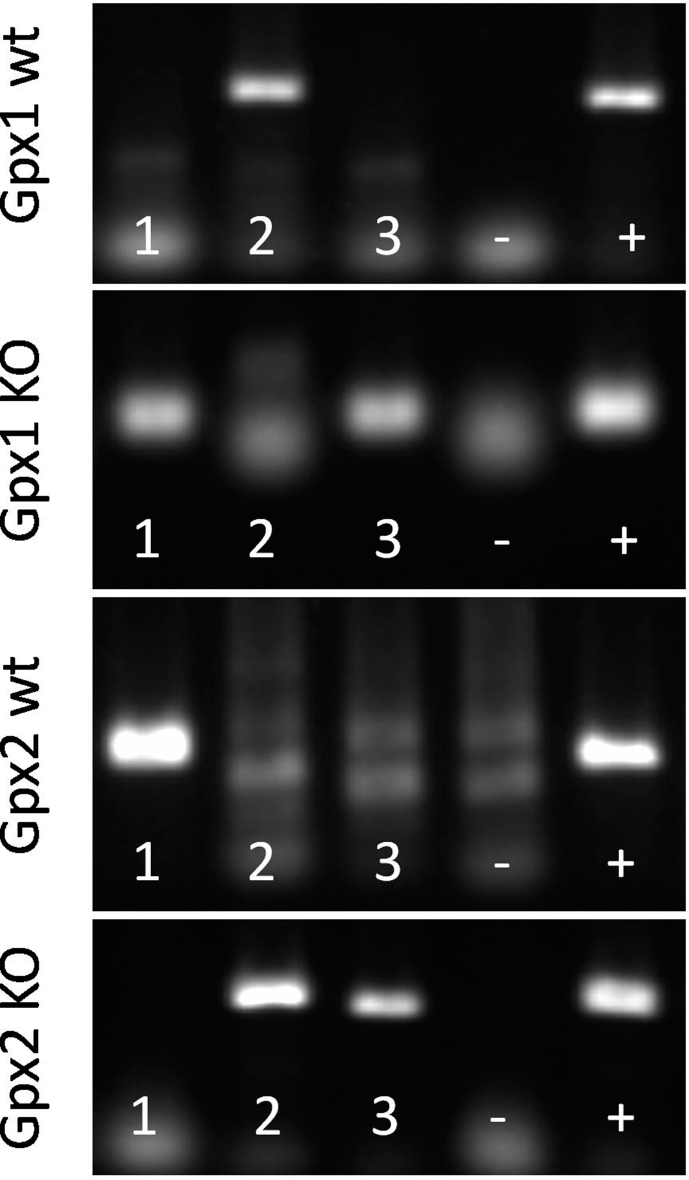
**Genotyping of GPX1 and GPX2 knockout mice.** The PCR results show the genotyping for GPX1 knockout (lane 1), GPX2 knockout (lane 2), and knockout of both of them (lane 3). For comparison, a positive (+) and negative (−) control sample was measured in parallel.

Wild type (WT) murine embryonic fibroblasts or MEFs with knockout of either GPX1 (G1KO), GPX2 (G2KO) or both (G1/2KO) were cultured in growth medium enriched with 50 nM selenite. While Gpx1 mRNA levels clearly confirmed the expected genotype of the cells (Fig. 1A), GPX2 expression was below the detection limit in all four cell lines both for qPCR (data not shown) and Western Blot (Fig. 1B). Therefore, the GPX2 knockout has been independently confirmed by genotyping (Fig. S1). GPX4 expression was maintained in cells cultured without additional selenium (Fig. 1B). Loss of GPX1 (either alone or in combination with GPX2) resulted in almost complete ablation of GPX activity towards H_2_O_2_, tBHP, HpETE, and HpODE (Fig. 1C). As GPX2 expression was almost undetectable in MEFs, genetic ablation of GPX2 expression had no effect on total GPX activity towards the four tested substrates (Fig. 1C). These results show that GPX1 provides the major part of hydroperoxide-reducing GPX activity in MEF cells, while GPX2 effects cannot be properly studied in this cellular system, indicating the limitations of available cellular systems to study both GPX enzymes beyond cancer cell lines. To identify substrate specificities of the two isoforms of GPX we next used human recombinant GPX preparations.

### 3.2 Characterization of the recombinant GPX proteins

**Fig. 2:**
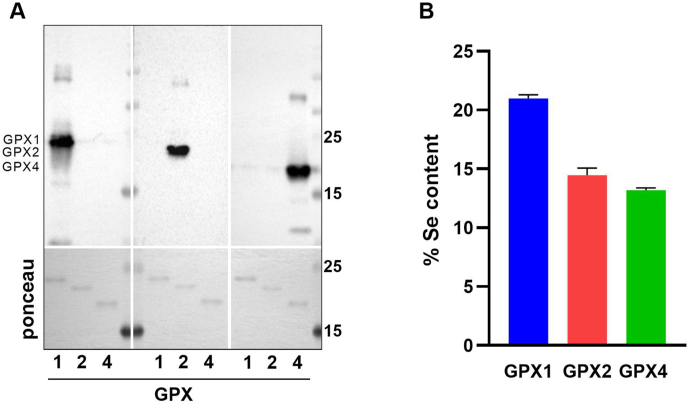
**Characterization of recombinant proteins.** Purity of recombinant human GPX1, GPX2, and GPX4 produced in *E.coli* was analysed using Western Blot. Ponceau staining was used to verify blotting efficiency (A). The selenium content of the recombinant proteins was measured using TXRF with 1 mg/l gallium as internal standard for 1000 s. The selenium content was calculated as percentage of selenium in relation to total protein concentration of each preparation. Data are depicted as mean + SD of five technical replicates.

First, we verified the purity and identity of the three recombinant GPX proteins as well as the specificity of the antibodies in Western blot analyses, which indeed showed the expected specific reactivities (Fig. 2A). Because not all recombinant selenoproteins will have the expected Sec content due to one-codon skipping or non-Sec mediated UAG suppression with glutamine or lysine [5,39] we assessed the selenium content of the recombinant proteins. This was measured using TXRF analysis, yielding 20, 14 or 13% for GPX1, GPX2 or GPX4, respectively (Fig. 2B), thus being comparable Sec contents to what has been described before [5,39]. In contrast to other GPX variants, which might have activity although Sec levels are reduced [47], only the Sec-containing variants have GPX activity synthesized in this *E.coli* system as described before [5,39]. Accordingly, the selenium content was used for calculations of final activity for each GPX preparation with the different substrates, as outlined in the Methods section.

### 3.3 NADPH consumption by GPX enzymes using different hydroperoxides

**Fig. 3:**
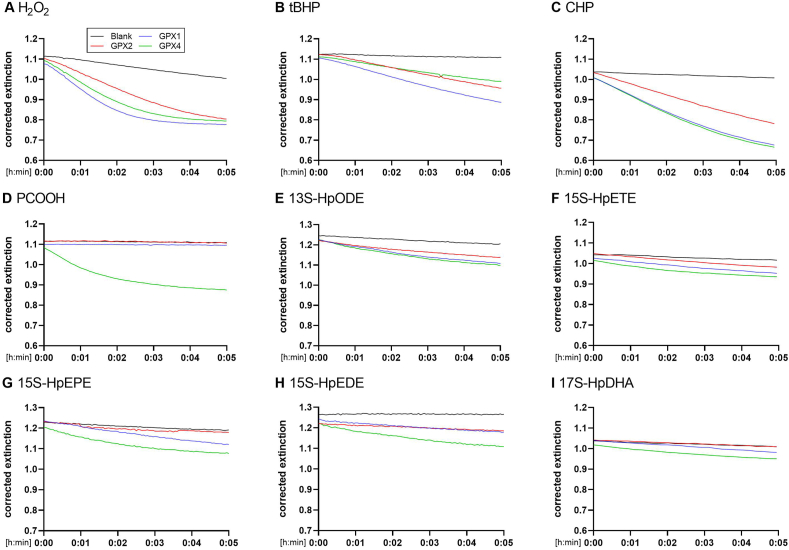
**Changes in NADPH consumption by GPX enzymes using different hydroperoxides.** 10 nM GPX1, 115 nM GPX2 or 120 nM GPX4 (48 nM GPX4 for PCOOH) were used to measure NADPH consumption after addition of 50 μM of various hydroperoxides over a period of 5 min at 340 nm. Data are depicted as mean of two or three technical replicates.

**Fig. S2:**
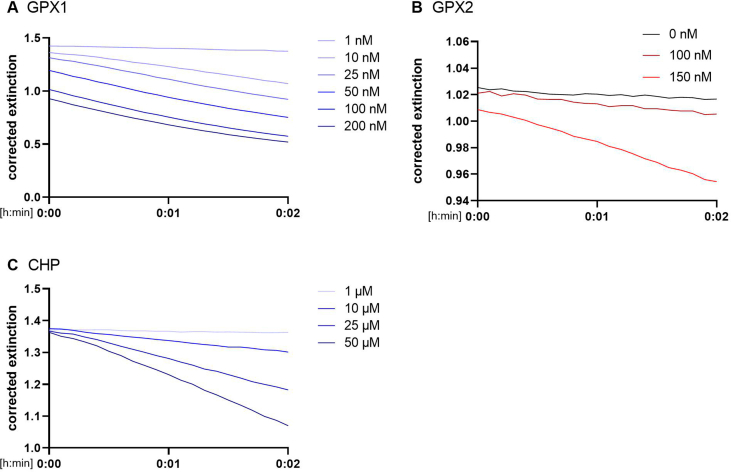
Changes in NADPH consumption of different amounts of enzyme or substrate. Different concentrations of GPX1 (A) or GPX2 (B) enzyme were tested in the standard GPX assay using 50 μM CHP as substrate. In addition, different amounts of CHP were reduced by using 10 nM GPX1 (C).

The GPX activity assay was standardized for all tested substrates using a final concentration of 50 μM of each substrate. The GPX enzyme concentrations were adjusted to obtain a linear reaction for at least 2 min. Representative curves for a range of GPX1 (Fig. S2A) and GPX2 (Fig. S2B) enzyme concentrations as well as different substrate concentrations for CHP (Fig. S2C) are shown. Accordingly, a total concentration of 10 nM GPX1, 115 nM GPX2 or 120 nM GPX4 (or 48 nM for the substrate PCOOH) were used as final concentrations for activity measurements (Fig. 3). For calculation of turnover numbers, the activities were subsequently normalized for Sec contents, as described above. We compared raw data of NADPH consumption measured at 340 nm during recordings not corrected for differences in protein amounts and selenium content of the proteins, because the concentrations of both substrates and enzymes were chosen in a way that good linear ranges and spans in 340 nm absorbance differences were obtained (for the subsequent calculation of selenium-correlated turnover numbers, see Methods section). The three simple hydroperoxides, H_2_O_2_, tBHP, and CHP could be efficiently reduced by all of the three GPX enzymes (Fig. 3A–C); in contrast, there was a clear substrate specificity for PCOOH for GPX4 with neither GPX1 nor GPX2 being able to reduce PCOOH under the conditions tested (Fig. 3D).

**Fig. S3:**
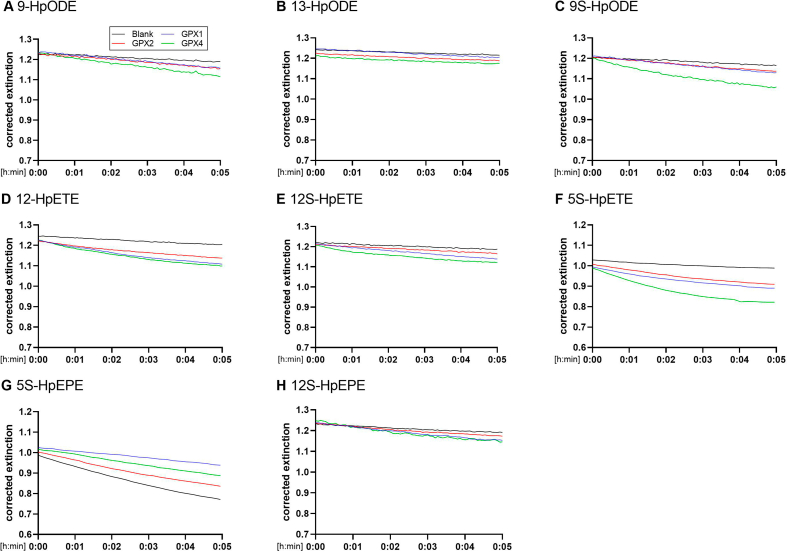
Changes in NADPH consumption of GPX isoforms using additional fatty acid-derived hydroperoxides. 10 nM GPX1, 115 nM GPX2 or 120 nM GPX4 (48 nM GPX4 for PCOOH) were used to measure NADPH consumption after addition of 50 μM of various hydroperoxides over a period of 5 min at 340 nm. Data are depicted as mean of two or three technical replicates.

Fatty acid-derived hydroperoxides are synthesized by lipoxygenases using different fatty acids, yielding products such as linoleic or arachidonic acid (Table 1). We thus tested 13 hydroperoxides derived from five different fatty acids for their susceptibilities to be reduced by GPX enzymes. All three enzymes were able to use the fatty acid-derived hydroperoxides as substrates, as shown here for one representative hydroperoxide for each fatty acid tested (Fig. 3E–I and S3). An exception was GPX2 towards hydroperoxides derived from eicosapentaenoic, eicosadienoic, and docosahexaenoic acid; very low activity was found with GPX2-catalysed reduction of 15S-HpEPE (Fig. 3G) and 17S-HpDHA (Fig. 3I) under the chosen assay conditions. In general, the fatty acid-derived hydroperoxides were also reduced less efficiently than the simple hydroperoxides by GPX1 and GPX2, while this difference was less pronounced for GPX4.

**Figure S4:**
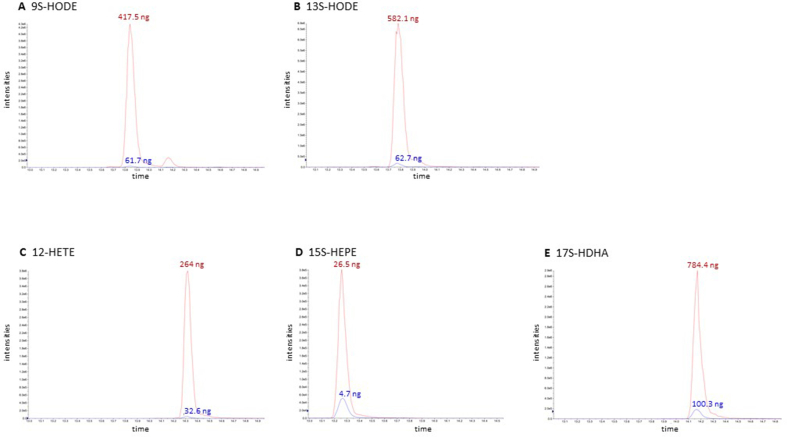
**Specificity of the GPX reaction towards production of single monohydroxyllated fatty acids derived from single hydroperoxides.** UPLC-MS/MS analysis was used to measure (A) 9S-HODE, (B) 13S-HODE, (C) 12-HETE, (D) 15S-HEPE, and (E) 17S-HDHA produced in a standard GPX assay using GPX1 as enzyme (red lines). In addition, the pure substrates were analysed as well (blue lines).

To test for the purity of the fatty acid-derived hydroperoxides, and further confirm the results obtained by the NADPH-coupled test, the corresponding monohydroxylated fatty acids of 9S-HpODE, 13S-HpODE, 12-HpETE, 15S-HpEPE, and 17S-HpDHA were quantified by UPLC. The respective chromatograms clearly indicated that the reaction products were detectable in the GPX reaction mixtures as expected. There was no difference in retention time between the different GPX isoforms and only the amount of the product differed, suggesting differences in overall activities but not in the reactions catalysed. Based on this, representative chromatograms are only shown for GPX1 reactions (Fig. S4, red lines). For 12-HETE, 15-HEPE, and 17-HDHA, only one specific peak was detectable (Figs. S4C-E). There was a small additional peak when measuring 9- and 13-HODE which was also detectable in the standard used as reference (Figs. S4A-B). Measuring only the substrates without adding GPX enzymes, only small amounts of the specific monohydroxylated fatty acids were detectable (Fig. S4, blue lines). This indicated that only through enzymatic conversion of corresponding peroxides by GPX activity high amounts of the specific monohydroxylated fatty acids could be obtained, as expected.

### 3.4 Enzymatic activity of the GPX enzymes calculated based on the amount of active selenoprotein

**Fig. 4:**
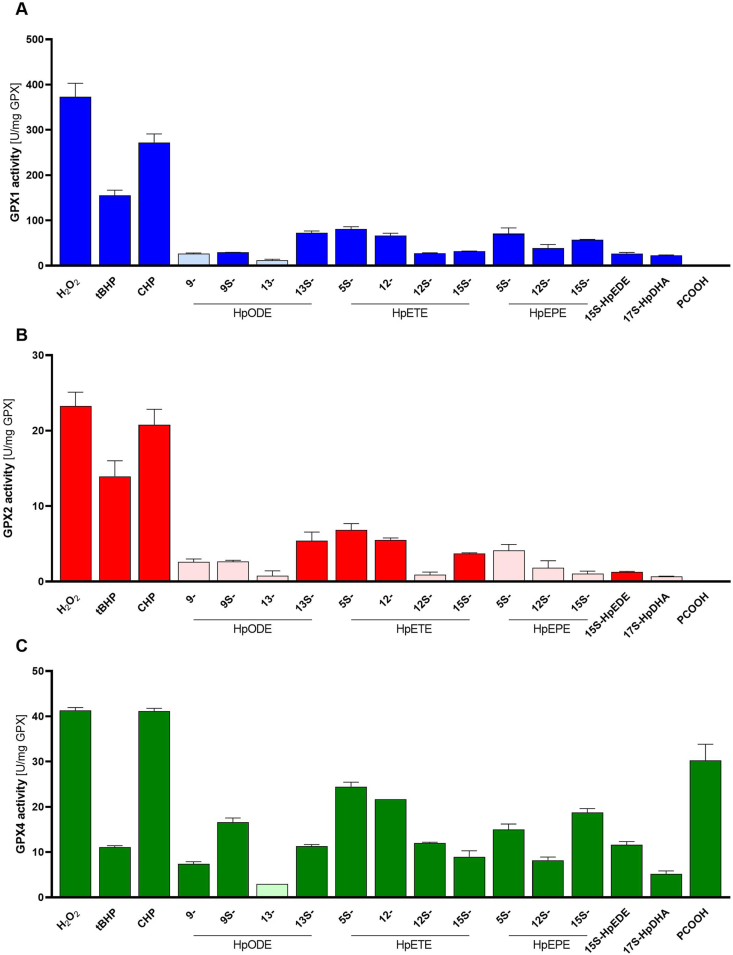
**Calculated enzymatic activity of GPX enzymes.** GPX activity of GPX1 (A), GPX2 (B), and GPX4 (C) was measured photometrically by a GR-coupled test and was calculated for each hydroperoxide based on the selenium content of the protein as U/mg GPX. Bars with more transparent colour indicate very low basal changes in NADPH consumption compared to blank values (< two times higher than blank). Data are depicted as mean + SD of two or three technical replicates. Significant differences between the GPX enzymes were calculated by two-way ANOVA revealing the following results: Differences between GPX1 and GPX2 were significant for all substrates except for PCOOH and 13-HpODE. Comparing GPX1 and GPX4 reached significance except for 9S-HpODE, 13-HpODE, 12S-HpETE, 15S-HpEDE. Significant differences between GPX2 and GPX4 were only observed for H_2_O_2_, CHP, 15S-HpEPE and PCOOH. (For interpretation of the references to colour in this figure legend, the reader is referred to the Web version of this article.)

To better compare the activities of the GPXs towards each tested substrate, the linear range in change of absorption of each NADPH consumption curve was used to calculate the corresponding enzyme activity normalized for selenoprotein contents. Overall, GPX1 showed the highest activity towards all the tested substrates, with about 10-fold higher activity than GPX2 and GPX4, except for the GPX4-specific substrate PCOOH (Fig. 4). For example, the GPX1-specific activity against H_2_O_2_ was 373 U/mg while it was 23.3 and 41.3 U/mg for GPX2 and GPX4, respectively. GPX2 showed almost exactly the same substrate specificity as GPX1 even though GPX2 was much less efficient than GPX1 (Fig. 4A and 4B). Differences between GPX1 and GPX2 were significant for all substrates except for PCOOH and 13-HpODE which were both close to the detection limit.

Only for GPX4, the pattern of substrate specificity substantially differed from the other two (Fig. 4C). As expected, GPX4 was the only isoform which could use PCOOH as substrate, but unexpectedly, the GPX4 activity towards H_2_O_2_ and CHP was in a comparable range than for PCOOH. In relation to H_2_O_2_, GPX4 had a higher relative substrate specificity towards the fatty acid-derived hydroperoxides than GPX1 and GPX2 (around 30–50% of H_2_O_2_-dependent activity in comparison to 10–20% for GPX1 or GPX2), but absolute turnover numbers were still much higher for GPX1 (e.g. for 5S-HpETE: 80.8 U/mg GPX1; 24.4 U/mg GPX4). The lowest GPX1 activity was measured against 17S-HpDHA which was 22.8 U/mg GPX1, but also this was higher than the corresponding GPX4 activity with 5.2 U/mg GPX4. Comparing GPX1 and GPX4 reached significance for all tested substrates except for 9S-HpODE, 13-HpODE, 12S-HpETE, 15S-HpEDE. In contrast, significantly higher activities of GPX4 in comparison to GPX2 were only observed for H_2_O_2_, CHP, 15S-HpEPE and PCOOH.

The linoleic acid-derived isomers of HpODE showed large differences regarding their reduction by GPX enzymes. While the racemic mixtures of 9-HpODE or 13-HpODE both displayed negligible activity with all three GPX enzymes (Figs. S3A and B), the reduction of 9S-HpODE or 13S-HpODE isomers was more efficient especially in the case of GPX4 (Fig. 4). In contrast, the racemic mixture of 12-HpETE was reduced more efficiently by the three GPX isoforms than the 12S-HpETE isomer (Fig. 4). In the group of fatty acid-derived hydroperoxides, the reductive capacity towards 5S-HpETE was the highest for all three enzymes. The activity of GPX2 against all three hydroperoxides derived from eicosapentaenoic acid (5S-, 12S-, and 15S-HpEPE) and the hydroperoxides derived from eicosadienoic acid (15S-HpEDE) and docosahexaenoic acid (17S-HpDHA) was very low. In contrast, both GPX1 and GPX4 were able to reduce these five substrates, and GPX1 again showed higher efficiency (Fig. 4).

## 4 Discussion

This study presents the first direct comparison of substrate specificities of GPX1, GPX2, and GPX4 with purified human enzymes. The results show that GPX1 and GPX2 have closely overlapping specificities, while GPX1 displays significantly higher turnover numbers than GPX2. GPX4 clearly reduced the complex lipid hydroperoxide PCOOH better than GPX1 and GPX2, but showed similar profiles of activity with H_2_O_2_, tBHP and CHP, although GPX1 again showed higher turnover than GPX4. These results help to shed further light on the possible roles of these three human selenoprotein GPXs *in vivo* and should be discussed in view of earlier findings using cellular and animal model systems.

Previous searches for specific substrates for either GPX1 or GPX2 using cellular systems have revealed no convincing results so far. Initially, GPX-overexpressing MCF-7 lines were studied indicating higher GPX activity towards all substrates in cells overexpressing GPX1 in comparison to GPX2 [37]. However, data were then not normalized to protein levels of the tested GPX enzymes. By directly comparing total GPX activity of HT-29 cells with stable shRNA-mediated knockdown of GPX1 or GPX2, a more pronounced reduction of total GPX activity was observed upon GPX1 downregulation. However, also in this model the knockdown efficiency differed between the lines resulting in 25% of residual GPX2 but only 8% of GPX1 in comparison to control cells [48]. Herein, we also used a cell model with MEFs isolated from GPX1 or GPX2 single or GPX1/2 double knockout mice as experimental model, which only gave direct obvious results for GPX1 knockout because the basal levels of GPX2 were hardly detectable in these fibroblast cells (Fig. 1). The total GPX activity towards H_2_O_2_ and tBHP was almost completely abolished in MEFs with loss of GPX1, which was also the case when using HpODE or HpETE as substrates, although starting from lower levels in WT. Thus, in MEF cell lysates, GPX1 is mainly responsible for reducing these lipid hydroperoxides even though GPX4 expression was clearly detectable. Thus, the MEF cells are limited in terms of studying GPX2 in comparison to GPX1. Also, other models are limited in this respect as e.g. in the murine intestine a knockout of GPX2 results in significantly higher total GPX activity in comparison to the WT animals which is mediated by upregulation of GPX1 [33], suggesting a direct cross-talk between GPX1 and GPX2 in cellular settings. Overall, this indicates that *in vivo* models based on mice or cell lines are limited in terms of studying distinct substrate specificities of GPX1 and 2 because of separate expression of both isoforms or compensatory effects taking place at the expression level.

Thus, our data are the first to directly compare human recombinant selenoproteins GPX1, GPX2, and GPX4 side by side in the standard NADPH-coupled GPX activity assay using a broad set of physiologically relevant hydroperoxide substrates. The chosen assay conditions are well in line with the standard GPX activity assay initially established and employed for decades [43,44]. As standard condition, NaN_3_ was added to the assay mixture which is not affecting GPX activity but specifically inhibits catalase activity. First, we tested different enzyme concentrations to achieve a linear curve during 2 min of measurement (Figs. S2A and B). For fatty acid hydroperoxides, up to 5 min could be used while the linear range was shorter for H_2_O_2_, CHP, or tBHP. Based on these initial measurements, almost 10-fold higher GPX2 and GPX4 concentrations had to be used in comparison to GPX1. These ratios were chosen to optimize *in vitro* assay conditions and are not related to ratios of enzymes within cells, which, however, depend very much on the cellular system, intracellular compartmentalization and the available selenium concentration. The substrate concentration was maintained at 50 μM which works best for stable assay performance. However, potential differences in critical micelle concentrations of the individual hydroperoxides could limit the comparability between the different substrates tested. The assay conditions ensure that GSH levels remain constant due to regeneration and the hydroperoxide concentration is close to the apparent V_max_ [44]. Previous studies showed that neither GPX1 from bovine blood, from hamster liver or from human erythrocytes had activity towards PCOOH, while GPX4 from pig heart had substantial activity [44]. We can here clearly confirm previous results suggesting that PCOOH is a specific substrate for GPX4 [49,50]. This specificity appears to be driven by the monomeric character of GPX4 in contrast to tetramers formed in case of GPX1 and GPX2 which results in less accessible binding pockets to the active side [51]. Aside from specificity for PCOOH, no substrate specificity could here be identified for any of the other tested 16 hydroperoxides, as all of them could be reduced to a certain extent by any of the three GPX enzymes. This finding contrast earlier claims that H_2_O_2_ is only a weak substrate for GPX4 [52], which is obviously not the case as H_2_O_2_ reduction by GPX4 was here found to be comparable with that of PCOOH (Fig. 4C). However, this could be different in the cellular setting as MEF lysates with GPX4 expression but loss of GPX1 showed almost no GPX activity towards H_2_O_2_ (Fig. 1). In addition, it has been proposed that GPX2 might have a higher prevalence for organic hydroperoxides than for H_2_O_2_ [37], which could not be confirmed by us. More specifically, cell culture experiments using HepG2 and CaCo2 cells with different selenium supply were used to identify substrate specificity of either GPX1 or GPX2. In selenium-deficient HepG2 cells with GPX2 expression but no detectable GPX1 expression, a substantial activity towards 13-HPODE could be determined which was interpreted as potential specific substrate for GPX2 [53]. However, our analyses reveal that even though the activity of all three GPX enzymes towards 13-HpODE was very low, GPX2 showed again the lowest activity (Fig. 4).

Our results raise questions regarding the biological roles and functions of the much less efficient GPX2 compared to GPX1, as the two enzymes have such similar substrate specificities while GPX1 is clearly much more efficient than GPX2. GPX2 can be described as an inducible enzyme because it is upregulated at the transcriptional level by Nrf2 [54], the major transcription factor activated in response to oxidative stress [55] while there is no clear data showing an Nrf2-dependent upregulation of GPX1 expression. However, at the translational level, GPX2 is maintained under selenium deficiency while GPX1 is drastically reduced [15]. Aside from that, GPX1 and GPX2 are expressed in different cell types. For example, in the intestinal epithelium GPX1 is mainly expressed in differentiated cells, while GPX2 is located at the crypt base [33] where stem cells reside that constantly divide. Under these circumstances the different turnover rates of both GPX enzymes might be of biological importance for fine tuning redox signalling. For example, in proliferating cells the hydroperoxide signal might need to be maintained a little bit longer [56] which can be achieved by high GPX2 expression acting as the main reducing enzyme instead of GPX1. Accordingly, the signal can be transmitted more efficiently, allowing for higher downstream signalling and maintained proliferation. The data provided here are not suitable to answer these questions but indicate that intra- and intercellular localization of GPX enzymes needs to be considered for proceeding further in understanding the specific function of GPX1 and GPX2 and to identify a potential interplay.

## Funding

This work was supported by the 10.13039/501100001659German Research Foundation (DFG) [FOR 2558] and the 10.13039/100007569Carl Zeiss Foundation (IMPULS). ESJA acknowledges funding from 10.13039/501100004047Karolinska Institutet, the Knut and Alice Wallenberg Foundations (KAW 2019.0059), the 10.13039/501100002794Swedish Cancer Society (21 1463 Pj), the 10.13039/501100004359Swedish Research Council (2021–02214), the Cayman Biomedical Research Institute (CABRI), the Hungarian Thematic Excellence Programme (TKP2021-EGA-44), the Hungarian National Research, Development and Innovation Office (ED_18-1-2019-0025), and the Hungarian National Tumor Biology Laboratory (02022–2.1.1-NL-2022-00010).

## Declaration of competing interest

The authors declare the following financial interests/personal relationships which may be considered as potential competing interests:As indicated in the manuscript, we hereby declare that QC and ESJA are working with and are shareholders of Selenzoyme AB, a company providing recombinant selenoproteins.

## Data Availability

Data will be made available on request.
